# Enhanced CNS transduction from AAV.PHP.eB infusion into the cisterna magna of older adult rats compared to AAV9

**DOI:** 10.1038/s41434-021-00244-y

**Published:** 2021-03-22

**Authors:** Diptaman Chatterjee, David J. Marmion, Jodi L. McBride, Fredric P. Manfredsson, David Butler, Anne Messer, Jeffrey H. Kordower

**Affiliations:** 1 Department of Neurological Sciences, Rush University Medical Center, Chicago, IL USA; 2 Parkinson’s Disease Research Unit, Department of Neurobiology, Barrow Neurological Institute, Phoenix, AZ USA; 3 Divison of Neuroscience, Oregon National Primate Research Center, Beaverton; Departments of Behavioral Neuroscience and Neurology, Oregon Health and Science University, Portland, OR USA; 4 Department of Translational Neuroscience, Michigan State University, Grand Rapids, MI USA; 5 Neural Stem Cell Institute, Regenerative Research Foundation, Rensselaer; Department of Biomedical Sciences, University at Albany, Albany, NY USA; 6 ASU-Banner Neurodegenerative Disease Research Center, Biodesign Institute, Arizona State University, Tempe, AZ USA

**Keywords:** Genetic vectors, Genetic transduction, Regeneration and repair in the nervous system

## Abstract

The development of high efficiency, central nervous system (CNS) targeting AAV-based gene therapies is necessary to address challenges in both pre-clinical and clinical investigations. The engineered capsids, AAV.PHP.B and AAV.PHP.eB, show vastly improved blood-brain barrier penetration compared to their parent serotype, AAV9, but with variable effect depending on animal system, strain, and delivery route. As most characterizations of AAV.PHP variants have been performed in mice, it is currently unknown whether AAV.PHP variants improve CNS targeting when delivered intrathecally in rats. We evaluated the comparative transduction efficiencies of equititer doses (6 × 10^11^vg) of AAV.PHP.eB-CAG-GFP and AAV9-CAG-GFP when delivered into the cisterna magna of 6–9-month old rats. Using both quantitative and qualitative assessments, we observed consistently superior biodistribution of GFP+ cells and fibers in animals treated with AAV.PHP.eB compared to those treated with AAV9. Enhanced GFP signal was uniformly observed throughout rostrocaudal brain regions in AAV.PHP.eB-treated animals with matching GFP protein expression detected in the forebrain, midbrain, and cerebellum. Collectively, these data illustrate the benefit of intracisternal infusions of AAV.PHP.eB as an optimal system to distribute CNS gene therapies in preclinical investigations of rats, and may have important translational implications for the clinical CNS targeting.

## Introduction

The use of adeno-associated viruses (AAV) for the targeting of the central nervous system (CNS) has revolutionized gene therapy for neurological disorders. However, AAV-vectors still face significant challenges in achieving broad and efficient CNS biodistribution and transduction. Although currently in clinical use, AAV9-mediated gene delivery requires high viral load to achieve relatively limited transduction throughout the CNS. Recent development of a Cre-dependent in vivo platform for the selection of target-specific AAV capsids led to the discovery of novel engineered AAV9-derived variants, AAV.PHP.eB and AAV.PHP.B [1, 2]. Both constructs remarkably enhanced the rate of viral blood-brain barrier (BBB) crossing when delivered peripherally in mice [1]. However, the pre-clinical efficiency of CNS targeting with both variants is highly variable depending on the animal strain [3], promoter selection [4], viral titer [1, 2, 5], or species [3, 6].

Significant evidence, primarily with AAV9, shows that more efficient global CNS penetration of AAVs can be achieved when delivered into the cerebrospinal fluid (CSF) [6–10]. Intrathecal delivery of AAV9 improves CNS targeting in both rodents and non-human primates (NHP) [6]. A direct comparison of intravascular and intrathecal delivery systems of AAV.PHP.B illustrated superior transduction profile from injections into the cisterna magna (CM) of NHPs than multiple intravascular injection modalities [6]. AAV.PHP capsids have primarily been characterized in mice, with relatively few data describing the capabilities of AAV.PHP variants in rats and rat-models of neurological disorders. One comparison of intravascular AAV.PHP.B-GFP and AAV.PHP.eB-GFP delivery in rats illustrated AAV.PHP.eB to be the superior vector for CNS targeting [5]. However, it remains unknown whether the optimal bio-distributive properties of AAV.PHP.eB in rats are maintained upon CSF delivery and how transduction efficiency compares to the well-characterized, and clinically utilized, AAV9 serotype.

Here, we aimed to address whether the AAV.PHP.eB virus improves CSF-mediated biodistribution in comparison to its parent serotype, AAV9, particularly for use in rat models of CNS disorders. We report significant enhancement of CNS viral transduction via delivery of AAV.PHP.eB into the CM of aged rats compared AAV9. Using a GFP reporter transgene to characterize viral biodistribution, we observed enhanced GFP expression consistently throughout neocortical and subcortical brain regions, including regions distal to the CM. Efficient transduction was also observed in regions without direct interface to CSF. We demonstrate significant increases in AAV.PHP.eB-mediated viral transduction compared to AAV9 with expected neuronal tropism and no indications of regional inflammation due to virus or transgene expression. These data demonstrate the value of using AAV.PHP.eB in CNS targeting for gene therapy studies in large rodents and highlight the potential of intracisternal (ICM) injections of this serotype clinically.

## Materials and methods

### Animals

All husbandry and procedural use of animals was approved by the Institutional Animal Care and Use Committee (IACUC) at Rush University Medical Center. Thirty middle-aged (6–9 month old), female Sprague–Dawley rats (Charles River Laboratories) were maintained at 25 °C and 50% humidity. Animals were housed in 12-hour light/dark cycle and fed chow and water ad libitum. All animals used in the study were acclimatized in the vivarium and handled for two weeks prior to initiation of study.

### Intracisternal injections

**Fig. 1:**
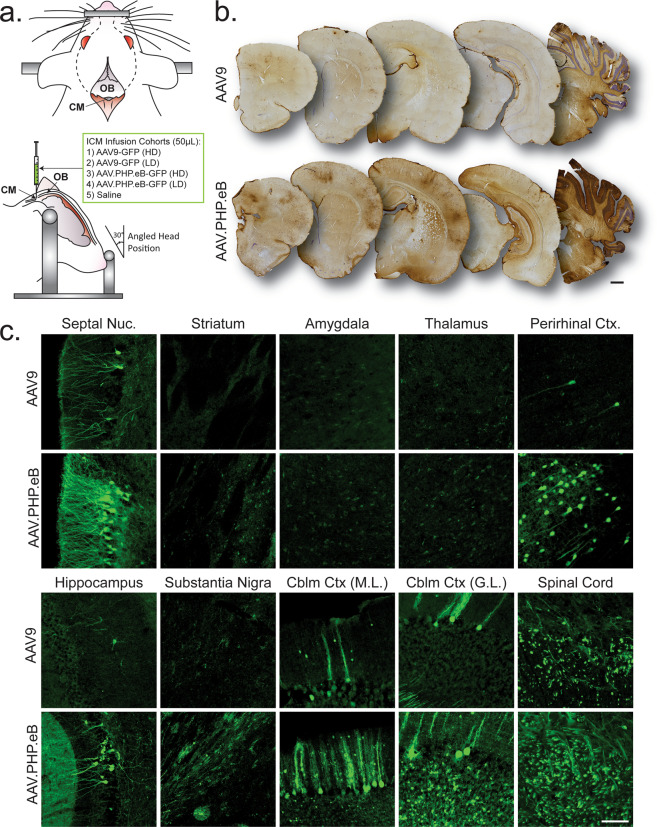
Expansive CNS transduction via intracisternal delivery of AAV.PHP.eB-GFP. **a** Visual schematic of intracisternal injection protocol for comparison of transduction efficiencies between AAV9 and AAV.PHP.eB. Middle-aged rats were loaded into stereotactic frames and oriented to elevate the occipital ridge and facilitate access to the cisternal membrane. 50 µL infusions of saline, AAV9 (high dose = 6 × 10^11^ vg/animal, low dose = 6 × 10^10^ vg/animal), or AAV.PHP.eB (high dose = 6 × 10^11^ vg/animal, low dose = 6 × 10^10^ vg/animal) were injected into the cisterna magna and animals were sacrificed 1-month post-injection. **b** Microphotographs of sections immunohistochemically labeled for GFP throughout the rostrocaudal extent of animals receiving high dose ICM injections of AAV9 or AAV.PHP.eB (Scale bar = 500 µm). **c** Confocal imaging of native GFP expression in AAV9 or AAV.PHP.eB-treated animals throughout various CNS regions of interest (Scale bar = 200 µm). *ML* molecular layer, *GL* granular layer.

Viral preps of AAV-CAG-GFP were a gift from Edward Boyden (Addgene viral prep #37825-AAV9, Addgene viral prep #37825-PHPeB; http://n2t.net/addgene:37825; RRID:Addgene_37825). Animals were anesthetized with sodium ketamine hydrochloride/xylazine hydrochloride solution (100 mg/kg and 10 mg/kg) and loaded into a stereotactic frame with the snout of the animal depressed by the nose bar to elevate the occipital crest (Fig. 1a). A two-centimeter incision was made and superficial connective tissue and midline muscles of the occipital crest were blunt dissected away until the atlanto-occipital membrane was exposed (Fig. 1a). A 50 µL syringe (Hamilton) with a Luer lock needle was plunged into the cisternal membrane until the bevel was completely submerged and a tight seal surrounding the needle was confirmed. Animals were infused with 50 µL of saline, AAV9-GFP (high-dose = 6 × 10^11^ vg, low-dose = 6 × 10^10^ vg), or AAV.PHP.eB-GFP (high-dose = 6 × 10^11^ vg, low-dose = 6 × 10^10^ vg) at an injection rate of 10 µL/min with a 10-minute post-injection needle dwell time (*N* = 6 for all five cohorts). At 1-month post injection, animals were deeply anesthetized with ketamine/xylazine cocktail and transcardially perfused with ice-cold PBS. Brains were removed and hemisected with one hemisphere immersion fixed overnight in 4% paraformaldehyde and the other hemisphere dissected to separate forebrain, midbrain, cerebellum, and spinal cord. CNS regions, as well as liver samples, were snap frozen and stored at −80 °C for molecular processing.

### Quantification of transduction analysis

Brains were sectioned and histologically processed as previously described [11] (detailed protocols are described in Supplementary Materials and Methods). Brightfield and confocal images were taken on an inverted confocal microscope (Nikon A1R). All quantitiation and analysis were performed blinded to treatment groups. Stereological estimates of GFP-labeled cells were performed on four distinct regions: motor cortex, somatosensory cortex, substantia nigra (pars compacta/pars reticulata), and cerebellum (lobule V/simple lobule) using Stereo Investigator software (MBF Bioscience, version 2019.1.2) on a brightfield microscope (Olympus BX50). Contours of three consecutive, level-matched sections for each region-of-interest were traced and GFP + cells were counted using the optical fractionator probe. Stereological estimates of microglia were performed on Iba1-labeled cells in the somatosensory cortex. Stereological assessment parameters for each region and summary data are described in Table S1. Analysis of regional staining was performed using ImageJ (NIH, version 1.52a). Tiled 10X microphotographs of level-matched sections for each subject were imaged at equivalent acquisition settings. Images were converted to 16-bit grayscale and positive staining was traced with a binary mask using thresholding. Each region-of-interest was outlined in congruence with the Allen Brain Atlas to determine the percentage of area stained. Viral tropism analysis was conducted using Cell Counter probe in ImageJ. Immunofluorescently labeled, level-matched images (7–9 per animal) across cortex, hippocampus, ventral midbrain, and cerebellum were acquired using a 20X lens. Brain cellular subtype markers that co-localized with native GFP expression were and labeled as neurons, oligodendrocytes, astrocytes, or non-specific cell types. Cellular tropism is reported as a percentage of total GFP + cells counted (minimum 50 cells/replicate). Densitometric analysis of GFP immunoblots was performed using ImageJ on images taken by an Odyssey near-IR scanner using equivalent acquisition settings (detailed immunoblot methods can be found in Supplementary Materials and Methods).

### Statistical analysis

All statistical analysis was performed using Prism software (GraphPad, Version 8.0.2). Stereological estimations and immunoblot data were analyzed with two-tailed, unpaired *T* test for normal data sets or a Mann–Whitney test for non-normal distributions. Comparisons of stereological estimates with more than two cohorts were analyzed via one-way ANOVA with a Tukey post-hoc test for multiple comparisons. Cellular tropism of AAV9 and AAV.PHP.eB-treated animals was analyzed via a two-factor ANOVA (treatment and cellular subtype) with Sidak’s post-hoc test for multiple comparisons. All data represented as Mean ± SEM with *p < 0.05 and **p < 0.01.

## Results

Here we report results from a rapid and streamlined intrathecal protocol that describes single-administration, high-volume dosing into the CM irrespective of adult animal age or weight (Fig. 1a). The methods used in this study do not require CSF-extraction prior to injection.

We first qualitatively evaluated the relative transduction profiles of AAV.PHP.eB-GFP and AAV9-GFP following equititer, high-dose ICM injection. Rats injected with AAV.PHP.eB-GFP showed significantly higher expression of GFP throughout the rostracaudal extent of the rat brain (Fig. 1b). GFP histology illustrates AAV.PHP.eB outperformed AAV9 in efficient transduction of cortical and subcortical layers with no notable loss of efficiency in rostral brain regions distal to the site of injection. Transduction is most notable in patterned clusters of GFP + perikarya observed throughout the cortex, spanning primarily through cortical layers III–V in AAV.PHP.eB-GFP-treated animals (Fig. 1b, c). By comparison, AAV9-GFP-treated animals feature sparse staining with minimal generalized fiber staining and greater GFP expression in the cerebellum and nuclei proximal to the CM (Fig. 1b, c). Peri-ventricular regions of AAV.PHP.eB-treated animals, such as the septal nuclei or ventral hippocampus, depict enhanced cellular staining (Fig. 1c). However, enhanced GFP + expression was not restricted to peri-ventricular regions, implying viral transduction efficiency is not solely a correlate of direct CSF contact (Fig. 1c). Cerebral and cerebellar cortical GFP expression is more prominent throughout AAV.PHP.eB-treated subjects, whereas subcortical regions (striatum/thalamus) feature high levels of GFP + puncta compared to that of AAV9-treated subjects (Fig. 1c). Increased AAV.PHP.eB-GFP expression can also be observed at both white and grey-matter regions along the lateral funiculus of the cervical spinal cord (Fig. 1c).

**Fig. 2:**
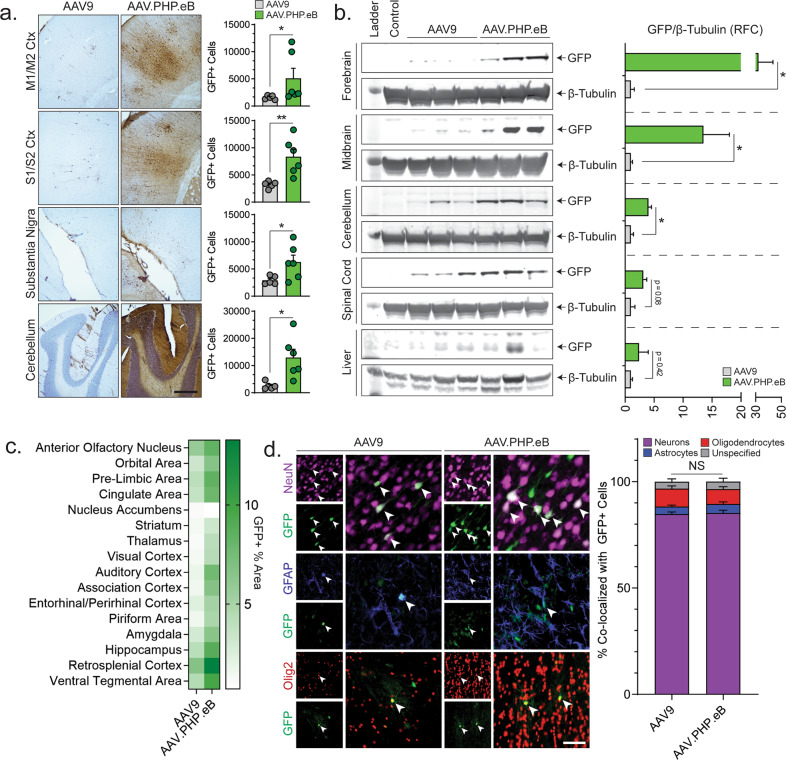
Quantitative analysis of enhanced biodistribution of AAV.PHP.eB vector compared to AAV9. **a** Representative images of DAB GFP labeling in the motor cortex (M1/M2), somatosensory cortex (S1/S2), substantia nigra, and cerebellum and corresponding stereological estimates of GFP+ cell populations (*n* = 5-6, scale bar = 500 µm). **b** GFP expression was analyzed across distinct biological replicates by immunoblot in forebrain, midbrain, cerebellum, spinal cord, and liver lysates. β-tubulin was used as an internal loading control. Densitometric analysis shows relative fold change of GFP/β-tubulin ratios (*n* = 3). **c** Heat map depicting mean percentage of area stained positively for GFP throughout regions of the rat CNS as a percentage of total outlined region area (*n* = 5-6). **d** Representative confocal microphotographs of native GFP+ expression in cells triple labeled with markers for neurons (NeuN), astrocytes (GFAP), or oligodendrocytes (Olig2). Arrows depict cells colocalized with GFP− transduced cells. Corresponding graph of cellular tropism determined by co-localization analysis. Data are represented as percentage of cellular subtype transduced/total GFP+ cells counted. All data represented as Mean ± SEM. Statistics: All *p < 0.05, **p < 0.01.

To quantify AAV.PHP.eB- and AAV9-mediated biodistribution, unbiased stereological estimates of GFP + neurons were performed across four primary regions (motor cortex, somatosensory cortex, substantia nigra, and cerebellum). High-dose AAV.PHP.eB significantly improved cerebellar transduction compared to high-dose AAV9 (+505%, *p* = 0.016), low-dose AAV.PHP.eB (+291%, *p* = 0.043), and low-dose AAV9 (+508%, *p* = 0.011) (Figs. 2a, S1a). Linear regression analysis of animal body weight showed a moderate inverse correlation with cerebellar transduction in high-dose AAV.PHP.eB-treated animals, but a reverse correlation for low-dose-treated cohorts (Fig. S2b). All subsequent quantitative analysis was focused on direct comparison between the high-dose cohorts of AAV.PHP.eB and AAV9. Higher GFP+ cell populations were observed in the motor cortex (+322%, *p* = 0.017), somatosensory cortex (+260%, *p* = 0.008), and substantia nigra (+214%, *p* = 0.044) of AAV.PHP.eB-treated animals versus AAV9-treated.

To validate whether stereology corresponded with levels of GFP plasmid expression, immunoblots were performed from lysates of tissue across multiple regions. Immunoblots of the forebrain, midbrain, and cerebellum showed significantly higher levels of GFP expression in the AAV.PHP.eB-treated cohort compared to that of AAV9 (Fig. 2b). Densitometric analysis of immunoblots showed the relative ratio of GFP protein to Beta-tubulin to be ~32-fold higher in the forebrain (*p* = 0.049), ~14-fold higher in the midbrain (*p* = 0.049), and ~4-fold higher in the cerebellum (*p* = 0.011) in AAV-PHP.eB-treated rats (Fig. 2b). Cervical spinal cord lysates showed trends of higher GFP expression (*p* = 0.078) (Fig. 2b). Liver expression disparities were insignificant (*p* = 0.427), indicating transduction efficiency increases remain limited to the CNS and not peripheral tissue (Fig. 2b).

The use of ICM delivery may provide a platform for effective pan-CNS treatment of neurodegenerative diseases. To this end, we aimed to evaluate transduction levels across regions-of-interest pertinent to rodent models of Parkinson’s Disease (PD) [11, 12]. Sixteen brain regions associated with PD model pathology (excluding substantia nigra and motor cortex, which were analyzed by stereology) were evaluated for extent of GFP+ staining (Fig. 2c). Across each investigated region, AAV.PHP.eB showed significantly higher levels of positively stained areas. To establish whether ICM injections of AAV.PHP.eB alters CNS tropism, colocalization analysis was performed with GFP+ cells that were stained with markers for neurons (NeuN), astrocytes (GFAP), and oligodendrocytes (Olig2). Tropic profiles between cohorts showed no significant differences in relative distribution of transduced cells %GFP colocalized with (1) neurons—AAV9: 84.8%, AAV.PHP.eB: 85.3%; (2) astrocytes—AAV9: 3.5%, AAV.PHP.eB: 4.2%; (3) oligodendrocytes—AAV9: 8.4%, AAV.PHP.eB: 7.0% (Fig. 2d). To confirm that ICM delivery of high-volume vectors did not elicit an inflammatory response, microglial recruitment was evaluated by stereological estimations (Fig. S2a). No changes were observed in the number of microglia in the somatosensory cortex of AAV9 or AAV.PHP.eB-injected animals compared to animals injected with ICM saline (Fig. S2a). Evaluation of microglia surrounding positively transduced cells showed no gross morphometric changes or density levels, indicating the absence of a reactive innate immune response in regions of viral transduction (Fig. S2b).

## Discussion

The generation of AAV.PHP.B and AAV.PHP.eB, as well as recent advances in capsid engineering [13, 14], has been an important step in illustrating how viruses can be modified to enhance BBB crossing and understanding associated mechanisms for viral transport [1, 2]. Several lines of evidence have shown that intrathecal delivery of viral vectors can enhance CNS penetration and optimize the ratio of CNS/peripheral transduction [6, 15, 16]. Intrathecal delivery of AAV9-gene therapies has been employed in clinical trials for the treatment of giant axonal neuropathy [7] and spinal muscular atrophy [17]. Although infusions into the CM are not frequently used in clinical practice for procedural risk of pontomedullary tissue damage compared to cauda equinal lumbar puncture, ICM injections provide direct access to central CSF flux and brain entry, particularly in small animal models in which CSF fluid dynamics hinder therapeutic diffusion [18]. Also, ICM injections are less invasive and can accommodate significantly higher-volume infusions than intracerebroventricular injections.

In this study, we evaluated a head-to-head comparison of equititer doses of AAV9 and AAV.PHP.eB to determine whether the superior CNS transduction phenomena observed in AAV.PHP.eB-injected mice were maintained in rats. Using a CAG promoter to drive GFP expression in both vectors, we observed superior transduction throughout the brain of AAV.PHP.eB-infused animals. Transduced soma were a prominent source of the increased AAV.PHP.eB biodistribution, as determined by stereological analysis and observed densities of GFP+ parikariya. However, visual inspection of AAV.PHP.eB-treated rat brains revealed marked increases in neuritic GFP expression, indicating a significant fraction of GFP-stained tissue sourced from neuronal processes compared to that of AAV9. Higher GFP staining was also detected in the neuropil surrounding the areas such as molecular layer of the dentate gyrus.

We incorporated several factors into our study to address the challenges in high-fidelity neurological disease modeling and intervention. First, viral penetration across the BBB is less effective in adult subjects compared to neonatal rodents [19–21]. We used older (6–9 months) rats to ensure that biodistribution would be consistent for age-dependent model paradigms. Similar studies investigating AAV9 and AAV.PHP variants have typically utilized young-adult (<2 months old) rodents [4, 5] that may accommodate more promiscuous viral transduction. Second, high-titer doses of intravascular AAV required to achieve effective CNS tropism have been reported to induce toxic peripheral effects and neurodegeneration in various animal models, thus warranting investigation of optimal dosing schema for AAV therapies [22]. Here, the dosages of AAV.PHP.eB administered in our high titer cohorts (6 × 10^11^ vg/animal) were ~3 times lower than the minimum doses utilized in comparable rat studies investigating intravascular delivery [4, 5]. Intrathecal AAV.PHP.eB at that dosage was effective in expansive CNS transduction, but did not show parallel significant increases in peripheral GFP expression in liver lysates. Other studies have shown mixed results in relative peripheral tissue tropism by AAV.PHP variants [3, 6]. We also were unable to detect any evidence of microglial activation as a result of either AAV9 or AAV.PHP.eB treatment, suggesting the dosages used in this study induced negligible innate-immune response to either capsid or viral load. In addition, a primary interest for vector biodistribution studies is to understand whether AAV candidates are optimal for treating designated areas vulnerable to neuropathological perturbation. Intraparenchymal injections of pre-formed fibrils of the protein alpha-synuclein is a common model-induction paradigm for Parkinson’s disease-related neurodegeneration. Thus, we focused our quantitative indices of GFP+ expression in areas with the highest incidence of pathology in rodent models to ultimately develop neuroprotective gene therapies and distribute them to anticipated regions of insult [11, 12]. The level of transduction in AAV.PHP.eB-treated animals was at least double than that of AAV9-treated animals in every region with the exception of the nucleus accumbens, with the most prominent increases (>3-fold) observed in the association cortex, auditory cortex, thalamus, visual cortex, and piriform area.

The enhanced BBB transport mechanism for AAV.PHP vectors has recently been linked to the GPI-tethered receptor lymphocyte antigen six complex (locus A), Ly6A [23, 24]. The discovery of Ly6A was a result of probing genetic factors that governed the rate of AAV.PHP variant BBB crossing in various strains of mice. Although both AAV.PHP capsids utilize Ly6A as an endothelial surface receptor, it is uncertain why AAV.PHP.eB outperforms AAV.PHP.B in both mouse [1] and rat CNS targeting [5]. Similarly, although our study highlighted the superiority of AAV.PHP.eB CNS tropism in rats, it is mechanistically unclear why AAV.PHP.eB transduction is improved to that of AAV9 in rat CSF delivery. Parenchymal transfer of viral particles through CSF-interstitial interfaces or receptor binding and intracellular shuttling through endosomes may be enhanced by the AAV.PHP.eB capsid. It is also difficult to establish what fraction of CSF injected virus is transferred to the blood supply as a result of subarachnoid CSF-blood transepithelial perfusion. We speculate that exchange of viral load into the vasculature contributes in-part to superior AAV.PHP.eB prevalence throughout the brain. Expression of homologous Ly6 superfamily members in rats may sufficiently permit enhanced AAV.PHP.eB transcytosis. Although Ly6A was identified amongst high-impact variants essential to BBB-crossing in murine studies [23, 24], other signature genomic sequences may contribute to AAV.PHP.eB-mediated CNS tropism in rats independent of Ly6 homologs. Modified from AAV9, AAV.PHP.eB also maintains specificity for endothelial galactose saccharides [25] and the universal AAV receptor (AAVR) [26] as binding partners for BBB crossing. Further studies to investigate routes of engineered vector CNS entry and are necessary to understand these phenomena and their translational relevance for clinical use.

Although AAV.PHP.eB exhibited improved CNS transduction efficiency compared to AAV9, ICM viral diffusion resulted in moderate levels of intra-cohort variability in GFP expression. One potential source of variability may have been the use of equivalent volumes of injectant across animals, regardless of weight. However, linear regression analyzing cerebellar GFP+ cell estimates and body weight showed conflicting data between high-dose and low-dose cohorts on whether animal size is a critical factor for predicting biodistribution. Use of animal brain, weight, or CSF volume may provide better standards for comparison to predict biodistribution for follow-up studies. Although AAV.PHP.eB improved transduction efficiency via ICM injection over AAV9, the improvement was modest compared to efficiency spikes observed through intravascular comparisons in mice [1]. In addition, similar to previous reports [15], only a marginal fraction of astrocytes and oligodendroglia were transduced by AAV.PHP.eB, even in conjunction with a universal CAG promoter, thus limiting the utility of AAV.PHP.eB for gliotropic targeting. Recent reports have described how capsid-promoter interactions can influence CNS tropism [27] and may require investigations into how promoter selection may alter AAV.PHP.eB cellular targeting. An evaluation of higher titers may be necessary to determine the peak transduction benefits of intrathecal AAV.PHP.eB administration. One procedural modification that may improve viral CNS entry is to employ Trendelenburg inversion (placing rodents in supine position with lower feet elevated 30 degrees above the head) for up to 2 h during vector infusion, which has been shown to dramatically enhance AAV9 distribution in rat brains [15].

Intrathecal administration of AAV.PHP.eB successfully achieves widespread transduction throughout the neuraxis with a simplified ICM injection paradigm that can be used in older rats with relatively low viral titers and minimal risk of adverse effects on subjects. These data show the relative utility of AAV.PHP.eB vectors compared to AAV9 for development of gene therapies in rat models of neurodegenerative diseases, with potential for more effective clinical therapeutics.

## Supplementary information

SUPPLEMENTARY INFORMATION - Enhanced CNS transduction from AAV.PHP.eB infusion into the cisterna magna of older adult rats compared to AAV9
