# Supplementary material for: Enhanced CNS transduction from AAV.PHP.eB infusion into the cisterna magna of older adult rats compared to AAV9
Source: Gene Ther. 2021 Mar 22;29(6):390–7. doi: 10.1038/s41434-021-00244-y (PMC9203269; doi:10.1038/s41434-021-00244-y)
Supplement: Supplementary file 1 — SUPPLEMENTARY INFORMATION - Enhanced CNS transduction from AAV.PHP.eB infusion into the cisterna magna of older adult rats compared to AAV9 [file 41434_2021_244_MOESM1_ESM.pdf]

SUPPLEMENTARY INFORMATION

Enhanced CNS transduction from AAV.PHP.eB infusion into the cisterna magna of older adult rats compared to AAV9

Diptaman Chatterjee[1], David J. Marmion[2], Jodi L. McBride[3], David Butler[4], Anne Messer[4], Jeffrey H. Kordower[1]

[1]Department of Neurological Sciences, Rush University Medical Center, Chicago, IL 60612

[2]Parkinson's Disease Research Unit, Department of Neurobiology, Barrow Neurological Institute, Phoenix, AZ 85013

[3]Divison of Neuroscience, Oregon National Primate Research Center, Beaverton, OR 97006; Departments of Behavioral Neuroscience, Oregon Health and Science University, Portland, OR 97239

[4]Neural Stem Cell Institute, Regenerative Research Foundation, Rensselaer, NY 12144; Department of Biomedical Sciences, University at Albany, Albany, NY 12208

Correspondence to:

Jeffrey H. Kordower, Ph.D.

Department of Neruological Sciences

Rush University Medical Center

1735 West Harrison Street

Chicago, IL 60612

312-563-3585 (tel)

312-563-3571 (fax)

jkordowe@rush.edu

SUPPLEMENTARY MATERIALS AND METHODS

SUPPLEMENTARY FIGURES

SUPPLEMENTARY TABLES

SUPPLEMENTARY MATERIALS AND METHODS

Immunohistochemistry and Immunofluorescence

Brains were sectioned on a sliding, frozen-stage microtome at 40 um intervals. Free-floating sections were quenched of endogenous peroxidase activity in sodium metaperiodate, blocked in 5% BSA and normal serum, and incubated overnight with primary antibodies for GFP (Living Colors, 632380, RRID: AB_10013427, 1:500) or Iba1 (Wako Chemicals, 019-19741, RRID: AB_839504, 1:1000). Primaries were washed off and tissue was incubated with biotinylated secondary antibodies (Vector Laboratories, BA-1000/BA-2000, RRID: AB_2313606/AB_2133581, 1:500) for one hour and avidin-biotin solution for 75 minutes. Staining was developed with 3,3'-diaminobenzidene (DAB) and H_2O_2, and mounted onto gelatin-coated slides and counterstained with cresyl violet. Immunofluorescent staining of tissue was performed with aforementioned protocols for blocking step and incubated with a primary cocktail of NeuN (Millipore, MAB377, RRID: AB_2298772, 1:1000), GFAP (Abcam, ab4674, RRID: AB_304558, 1:2000), Olig2 (Abcam, ab109186, RRID: AB_10861310 1:500), or individually labeled with Iba1 (Wako Chemicals, 019-19741, RRID: AB_839504, 1:500). Primary antibodies were washed off and tissue was incubated with corresponding secondary antibodies raised against primary-antibody host species (Jackson ImmunoResearch, DyLight 405 AffiniPure Donkey Anti-Chicken, 703-475-155, RRID: AB_2340373, 1:500; Invitrogen, Alexa Fluor 555 Donkey Anti-Rabbit, A-31572, RRID: AB_162543, 1:500; Invitrogen, Alexa Fluor 647 Donkey Anti-Mouse, A-31571, RRID: AB_162542, 1:500) and mounted onto gelatin-coated slides. All mounted sections were dehydrated in ascending concentrations of ethanol, cleared with xylene, and cover slipped.

Immunoblotting

Dissected brain regions were homogenized in RIPA buffer (50mM Tris HCl, 150mM NaCl, 1% Triton-X, 0.5% Sodium Deoxycholate, and 0.1% SDS) containing protease and phosphatase inhibitors (Halt). Homogenates were lysed for 30 minutes and centrifuged at 16000rpm for 20 minutes at 4°C. Supernatant was extracted and protein concentrations were determined using a BCA assay (Bio-Rad). 20ug of protein was resolved via SDS-polyacrylamide electrophoresis (4-12% gradient gels) and transferred to nitrocellulose membranes. Membranes were blocked for one hour (Li-Cor) and incubated with primary antibodies overnight (GFP, Living Colors, 632380, RRID: AB_10013427, 1:1000; β-Tubulin, Abcam, ab8227, RRID: AB_2305186, 1:5000). Blots were washed an incubated with secondary antibodies conjugated to IRDyes (Li-Cor, IRDye 800CW Goat anti-Mouse IgG, 926-32210, RRID: AB_621842; Li-Cor, IRDye 680RD Donkey anti-Rabbit IgG, 926-68073, RRID: AB_10954442) and imaged with an Odyssey scanner.

SUPPLEMENTARY FIGURES

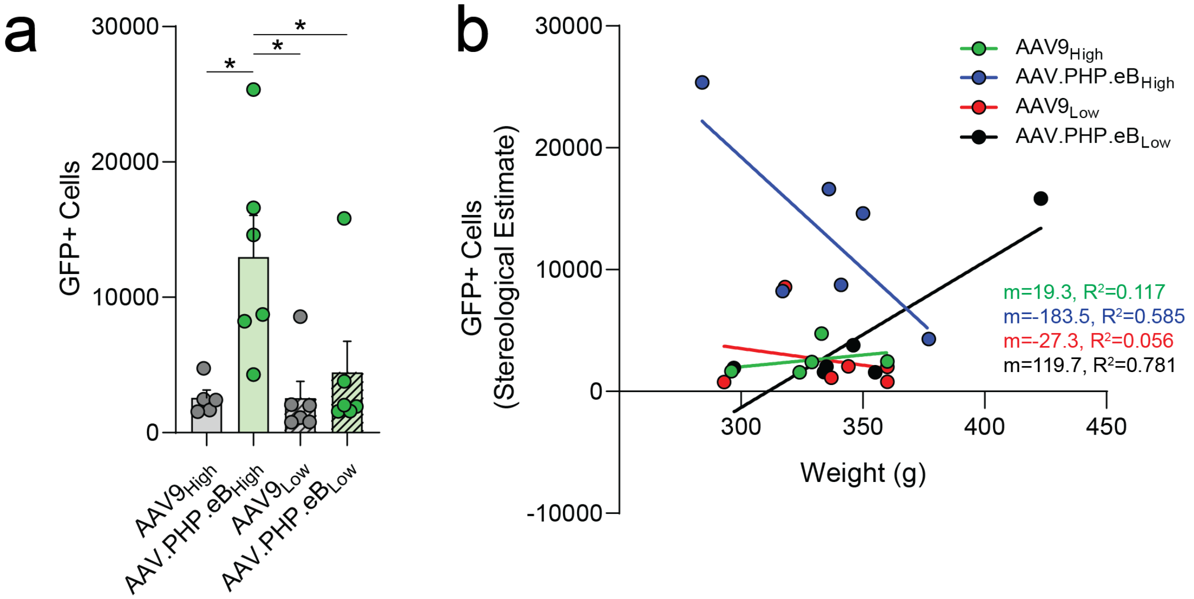

Figure S1. Stereological comparison of high- and low-titer AAV.PHP.eB-GFP and AAV9-GFP in the cerebellum. Middle-aged (6-9 month old) rats received intracisternal infusions of high-titer (6 x 10^{11} vg/animal) and low-titer (6 x 10^{10} vg/animal) AAV.PHP.eB-GFP or AAV9-GFP and were sacrificed one-month post-injection. **A)** Stereological estimations of GFP labeled cells in lobule V and the simple lobule of the cerebellum (n=5-6). **B)** Linear regression analysis of GFP+ cells as a function of animal body weight. All data represented as Mean ± SEM. Statistics: All *$p<0.05$, **$p<0.01$.

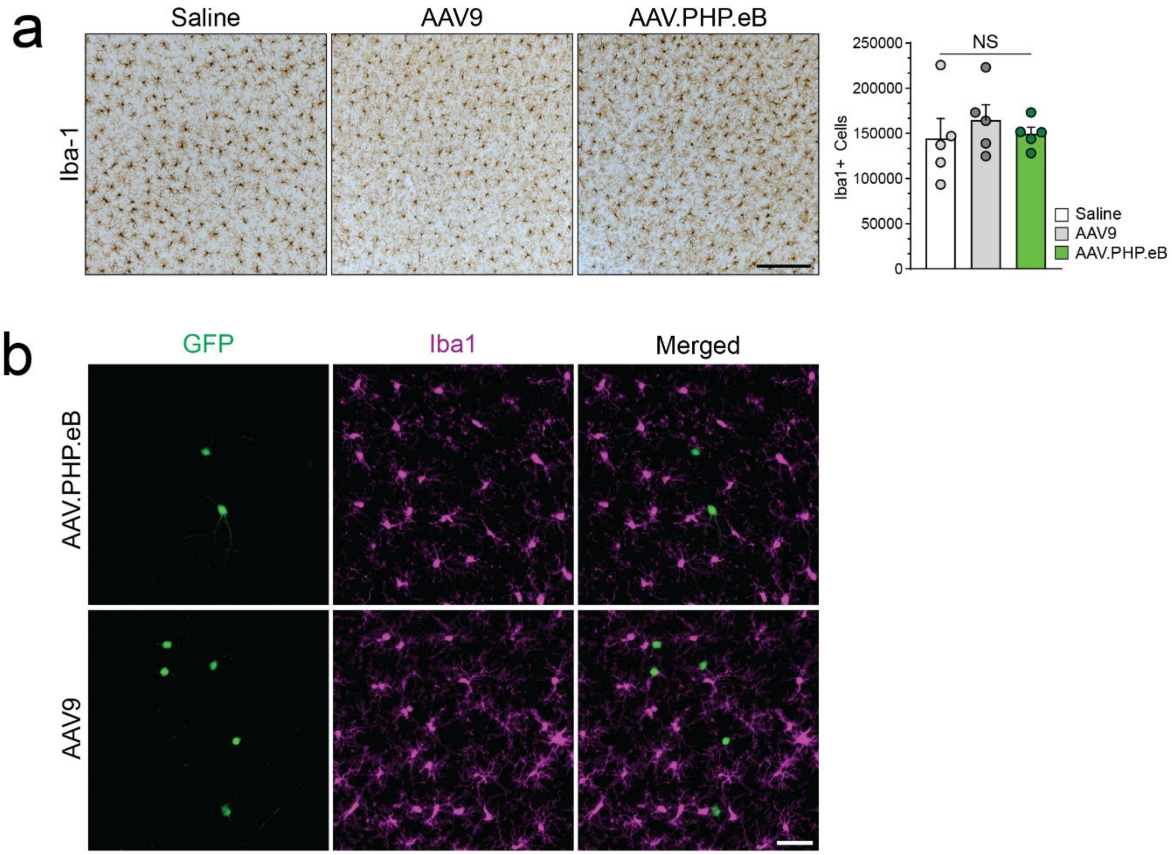

Figure S2. Evaluation of microglial activity and recruitment in response to AAV.PHP.eB-GFP or AAV9-GFP transduction. Iba1+ cells indicative of microglia were labeled to evaluate innate immune response to viral transduction. **A)** Representative images of Iba1+ cells in the somatosensory cortex of animals treated intracisternal saline, AAV9-GFP, or AAV.PHP.eB and corresponding stereological estimates of microglial population in each cohort (n=5, scale bar = 200µm). **B)** Immunofluorescent images of native GFP expression and Iba1+ microglia in AAV9-GFP- and AAV.PHP.eB-GFP-treated animals (scale bar = 50µm). All data represented as Mean ± SEM. Statistics: ns = not significant.

SUPPLEMENTARY TABLES

Marker	Region	Cohort	Counting Frame Area (µm²)	Sampling Grid Area (µm²)	Sampling Percentage	Section Interval	Estimated Population (Mean ± SEM)	CE (Gunderson, m=0)	CE (Gunderson, m=1)
GFP	Cerebellum	AAV9 (Low Dose)	4900	97969	5%	6	2555 ± 501	0.39	0.33
		AAV9 (High Dose)					2570 ± 258	0.27	0.25
		AAV.PHP.eB (Low Dose)					4457 ± 939	0.31	0.25
		AAV.PHP.eB (High Dose)					12974 ± 1258	0.21	0.14
	Motor Cortex	AAV9 (High Dose)	4900	97969	5%	12	1584 ± 72	0.32	0.32
		AAV.PHP.eB (High Dose)					5106 ± 755	0.27	0.22
	Somatosensory Cortex	AAV9 (High Dose)	4900	97969	5%	12	3220 ± 126	0.35	0.32
		AAV.PHP.eB (High Dose)					8370 ± 556	0.23	0.21
	Substantia Nigra	AAV9 (High Dose)	4900	97969	5%	12	2931 ± 155	0.38	0.34
		AAV.PHP.eB (High Dose)					6278 ± 515	0.29	0.26
Iba1	Somatosensory Cortex	Saline	4900	490000	1%	24	144319 ± 9964	0.23	0.16
		AAV9 (High Dose)					164825 ± 7576	0.22	0.16
		AAV.PHP.eB (High Dose)					149688 ± 3234	0.21	0.16

Table S1. Parameters for stereological estimations of GFP- and Iba1- labeled cell populations.
